# Yeast Culture Alleviates High-Concentrate Diet-Induced Rumen Fermentation Disturbances and Modulates the Rumen Microbiota and Metabolome in Lambs

**DOI:** 10.3390/microorganisms14092091

**Published:** 2026-09-18

**Authors:** Mingqiang Niu, Feifei Yang, Jiangfeng Fan, Shuru Cheng

**Affiliations:** 1 College of Veterinary Medicine, Gansu Agricultural University, Lanzhou 730070, China; 2 Gansu Cattle and Sheep Embryo Engineering Technology Research, Lanzhou 730070, China; 3 College of Animal Science and Technology, Gansu Agricultural University, Lanzhou 730070, China

**Keywords:** lambs, high-concentrate diet, yeast culture, rumen, rumen microorganisms, metabolomics

## Abstract

This study evaluated whether yeast culture (YC) supplementation could alleviate rumen disturbances induced by a high-concentrate diet in lambs. Forty-five three-month-old Du × Han F1 male lambs were assigned to control (CON), high-concentrate (HC), or HC supplemented with 0.5% YC groups (*n* = 15 each) for 60 days; six lambs per group were sampled for serum biochemistry, rumen fermentation, 16S rRNA gene sequencing, and untargeted metabolomics. Compared with CON, HC markedly reduced rumen pH (6.55 vs. 5.52) and the acetate-to-propionate ratio (2.67 vs. 1.31), whereas YC increased these values to 6.32 and 2.35, respectively (*p* < 0.05). YC also increased acetate and butyrate concentrations and reduced propionate relative to HC, while serum ALT returned to a level comparable to CON. YC partially reversed these fermentation changes and restored serum ALT to CON-comparable levels. YC also increased the Shannon index relative to HC and showed features relative to CON. LEfSe identified *g__Mitsuokella*, *g__Megasphaera_A_38685*, *g__Succiniclasticum*, *g__Parafannyhessea*, and *g__CAG-269* as candidate YC-responsive genera. Metabolomics identified 188 and 436 differential metabolites in CON versus HC and HC versus YC, respectively, mainly involving amino acid and cofactor metabolism. Several metabolites shifted toward CON levels, and correlation analysis linked candidate genera and metabolites with rumen fermentation and selected serum biochemical parameters. Overall, 0.5% YC partially alleviated high-concentrate diet-induced rumen acidification and was associated with improved microbial and metabolic homeostasis.

## 1. Introduction

As meat sheep production becomes increasingly intensive and large-scale, high-concentrate diets are widely used because of their high energy density and ability to meet the nutritional demands of rapidly growing animals. However, prolonged or excessive feeding of high-concentrate diets rich in rapidly fermentable carbohydrates can increase the availability of fermentable substrates in the rumen, thereby promoting the rapid production of volatile fatty acids (VFAs) and lactate [1]. When organic acid production exceeds the capacity for their absorption, utilization, and removal from the rumen, ruminal pH declines, increasing the risk of subacute ruminal acidosis (SARA) [2]. Moreover, a persistently low ruminal pH can alter the VFA profile and disrupt the composition and metabolic functions of the rumen microbial community, potentially impairing rumen barrier function and systemic metabolic homeostasis [3,4]. Rumen microorganisms constitute a critical interface between dietary composition and host nutrient metabolism. Through microbial fermentation, they convert dietary carbohydrates, proteins, and other nutrients into VFAs and a wide range of metabolites that provide essential energy and metabolic substrates for the host [5]. High-concentrate diets can reshape the rumen microbial community and its metabolic activity by altering substrate availability and the ruminal physicochemical environment, particularly pH [6]. Therefore, conventional fermentation parameters such as ruminal pH and VFA concentrations alone are insufficient to comprehensively characterize the biological changes induced by high-concentrate feeding. Integrating microbial community profiling with metabolomic analysis can provide a more comprehensive understanding of alterations in both microbial composition and metabolic function.

Yeast culture (YC) is a complex biological product derived from yeast fermentation. It contains yeast cells, enzymes, vitamins, and various fermentation-derived metabolites and has been widely used as a feed additive in ruminant nutrition [7]. Previous studies have shown that YC supplementation can improve the ruminal fermentation environment, modulate VFA profiles and microbial community composition, and influence microbial populations involved in lactate utilization, fiber degradation, and organic acid metabolism [8]. However, the effects of YC supplementation vary with diet composition, supplementation level, animal physiological status, and product characteristics, resulting in inconsistent responses in rumen fermentation and microbial community structure across studies [9]. In particular, under high-concentrate feeding conditions, whether YC can alleviate ruminal fermentation disturbances through coordinated changes in the rumen microbial community and metabolic profile remains unclear.

Although previous studies have reported the effects of yeast-based additives on rumen fermentation, microbial communities, or metabolic profiles, the coordinated responses of rumen fermentation, bacterial community structure, and rumen metabolites to yeast culture supplementation under high-concentrate feeding conditions remain insufficiently characterized. The novelty of the present study lies in the integrated evaluation of rumen fermentation, serum biochemical parameters, rumen bacterial communities, and untargeted metabolomic profiles within the same high-concentrate-fed lamb model, thereby providing new data on the coordinated microbial and metabolic changes associated with YC supplementation. Therefore, the aim of this study was to determine whether supplementation with 0.5% YC could attenuate ruminal acidification and fermentation disturbances induced by a high-concentrate diet in lambs and to characterize the associated changes in rumen bacterial communities and metabolic profiles. To address this aim, rumen pH, volatile fatty acids, serum biochemical parameters, 16S rRNA gene sequencing, and untargeted metabolomics were analyzed, and exploratory associations among selected bacterial genera, metabolites, rumen fermentation parameters, and serum biochemical indicators were further evaluated.

## 2. Materials and Methods

### 2.1. Animal Ethics Statement

All experimental procedures were approved by the Laboratory Animal Ethics Committee of Gansu Agricultural University (GSAU-Eth-AEW-2024-032) and were conducted in accordance with relevant ethical guidelines and regulations.

### 2.2. Experimental Animals and Sample Collection

A total of 45 healthy, three-month-old F1 male lambs from a Dorper (♂) × Small-tailed Han (♀) cross, with similar initial body weights and body condition, were used in the feeding trial conducted from October to December 2024 in Lingwu City, Ningxia Hui Autonomous Region, China. The lambs were randomly assigned to three dietary treatments, with 15 animals per treatment: a control group (CON; concentrate-to-forage ratio, 1.3:1), a high-concentrate group (HC; concentrate-to-forage ratio, 1.7:1), and a high-concentrate diet supplemented with 0.5% yeast culture group (YC; concentrate-to-forage ratio, 1.7:1). The lambs were housed in group pens under identical environmental and management conditions throughout the experiment.

The experimental diets were formulated with reference to the Chinese Agricultural Industry Standard NY/T 816-2021 [10], Nutrient Requirements of Meat-Type Sheep and Goat. All lambs were fed pelleted total mixed rations twice daily at approximately 08:00 and 18:00, with free access to clean drinking water. The yeast culture used in this study was a commercial product (Yi Su Kang; Xi’an Xinhanbao Biotechnology Co., Ltd., Xi’an, China) and was incorporated into the YC diet during feed processing before pelleting. The ingredient composition and nutrient levels of the experimental diets are presented in Table 1. The feeding trial lasted 60 days.

Body weight was recorded at the beginning and end of the feeding trial to determine initial body weight, final body weight, total weight gain, and average daily gain (ADG). Feed intake was recorded throughout the trial, and average daily feed intake (ADFI), feed conversion ratio (FCR), and gain-to-feed ratio (G:F) were calculated accordingly.

At the end of the feeding trial, six lambs were randomly selected from each treatment group for sample collection, providing six biological replicates per treatment for serum biochemical analysis, rumen fermentation measurements, 16S rRNA gene sequencing, and untargeted metabolomic analysis. The selected lambs were transported to a commercial slaughterhouse and fasted for 12 h before slaughter. Blood samples were collected from the jugular vein before slaughter and processed for serum preparation. The lambs were then electrically stunned and exsanguinated by severing the carotid arteries and jugular veins. Rumen contents were collected immediately after slaughter, with approximately 25 mL obtained from each lamb. Rumen pH was measured immediately after collection. Aliquots for 16S rRNA gene sequencing and untargeted metabolomic analysis were snap-frozen in liquid nitrogen and stored at −80 °C until analysis, whereas the remaining rumen content was stored at −20 °C for subsequent determination of volatile fatty acid (VFA) concentrations.

### 2.3. Measurement of Serum Biochemical Parameters

Whole blood samples were allowed to clot at room temperature for 2 h and then centrifuged at 3000 r/min for 15 min at 4 °C to obtain serum. The serum samples were aliquoted and stored at −80 °C until analysis. Serum biochemical parameters were measured using a Chemray 240/800 automated biochemical analyzer (Shenzhen Leidu Life Sciences, Shenzhen, China) with the corresponding commercial reagent kits. The measured parameters included total protein (TP), albumin (ALB), alanine aminotransferase (ALT), aspartate aminotransferase (AST), alkaline phosphatase (ALP), urea (UREA), uric acid (UA), glucose (GLU), triglycerides (TG), and total cholesterol (T-CHO). All measurements were performed according to the manufacturers’ instructions.

### 2.4. Determination of Rumen pH

Immediately after slaughter, rumen contents were collected and thoroughly mixed. Before measurement, a portable pH meter (Testo 205, Testo SE & Co. Titisee-Neustadt, KGaA, Germany) was calibrated using standard buffer solutions. The electrode was inserted directly into the thoroughly mixed rumen contents, and the pH value was recorded after the reading had stabilized. After each measurement, the electrode was rinsed with deionized water and gently dried before analysis of the next sample to minimize cross-contamination. All samples were analyzed as soon as possible after collection to minimize potential changes in rumen pH caused by exposure to air and temperature fluctuations.

### 2.5. Determination of Volatile Fatty Acids in Rumen Contents

Volatile fatty acid (VFA) concentrations were determined by gas chromatography (Agilent 8890, Agilent Technologies, Wilmington, DE, USA) using 2-EB as the internal standard, according to the method described by Shi et al. [11] with minor modifications. Rumen content samples were centrifuged at 5400 r/min for 10 min at 4 °C. Subsequently, 1 mL of the supernatant was transferred to a 1.5-mL centrifuge tube and mixed with 0.2 mL of 25% metaphosphoric acid solution containing 2-EB. The mixture was thoroughly vortexed, incubated on ice for 30 min, and then centrifuged at 10,000 r/min for 10 min at 4 °C. The resulting supernatant was filtered through a 0.22-μm membrane filter using a 2-mL syringe, and the filtrate was collected in a 2-mL amber autosampler vial for gas chromatographic analysis. The injector and detector temperatures were set at 250 °C and 260 °C, respectively. The oven temperature was initially maintained at 60 °C for 1 min, increased to 115 °C at 5 °C/min, and then increased to 180 °C at 15 °C/min.

### 2.6. 16S rRNA Gene Sequencing and Bioinformatics Analysis

Total microbial genomic DNA was extracted from rumen content samples using the OMEGA Soil DNA Kit (Omega Bio-Tek, Norcross, GA, USA). DNA integrity was assessed by 0.8% agarose gel electrophoresis, while DNA concentration and purity were determined using a NanoDrop 2000 spectrophotometer (Thermo Fisher Scientific, Wilmington, DE, USA). The V3–V4 hypervariable region of the bacterial 16S rRNA gene was amplified using the universal primers 338F (5′-ACTCCTACGGGAGGCAGCA-3′) and 806R (5′-GGACTACHVGGGTWTCTAAT-3′) containing sample-specific barcodes [12].

PCR amplicons were purified using the Agencourt AMPure XP kit (Beckman Coulter, Brea, CA, USA), after which sequencing libraries were constructed using the NEBNext Ultra II DNA Library Prep Kit (New England Biolabs, Ipswich, MA, USA) and subjected to paired-end sequencing (2 × 250 bp) on an Illumina sequencing platform. Raw paired-end reads were processed using Cutadapt (v4.9) to remove primer sequences and subsequently analyzed in QIIME 2 (v2024.2). The DADA2 plugin was used for quality filtering, denoising, paired-end read merging, and chimera removal to generate amplicon sequence variants (ASVs). Taxonomic assignment of ASVs was performed against the Greengenes2 database (release September 2024) using a confidence threshold of 0.8. The functional potential of the rumen microbial community was predicted using PICRUSt2 (v2.5.2) [13], and predicted functions were annotated against the Kyoto Encyclopedia of Genes and Genomes (KEGG) database. Alpha diversity was evaluated based on the Shannon diversity index and observed features using the ASV abundance table. Rarefaction curves based on observed features were generated to assess whether the sequencing depth was sufficient to capture bacterial richness. Beta diversity was evaluated using Bray–Curtis dissimilarity, and principal coordinate analysis (PCoA) was performed to visualize differences in bacterial community composition among the treatment groups.

### 2.7. Untargeted Metabolomic Analysis of Rumen Contents

Untargeted metabolomic profiling of rumen content samples was performed using ultra-high-performance liquid chromatography coupled with tandem mass spectrometry (UHPLC-MS/MS). Following sample preparation, data were acquired in both positive and negative ionization modes using an UHPLC system coupled to a quadrupole–Orbitrap high-resolution mass spectrometer (Orbitrap Exploris series, Thermo Fisher Scientific, Waltham, MA, USA). Quality control (QC) samples were prepared by pooling equal aliquots of all experimental samples and were analyzed periodically throughout the analytical sequence to evaluate instrument stability and data reproducibility.

Raw mass spectrometric data were processed for peak detection, extraction, alignment, and integration to generate a peak-area matrix. Data preprocessing included feature filtering based on relative standard deviation (RSD), missing-value filtering using the 50% rule, missing-value imputation using one-half of the minimum detected value, total ion current (TIC) normalization, logarithmic transformation, and mean centering.

The preprocessed data were imported into SIMCA software (version 18.0.1, Sartorius Stedim Data Analytics AB, Umeå, Sweden) for multivariate statistical analysis. Principal component analysis (PCA) was performed to evaluate the overall distribution and clustering patterns of the samples. Orthogonal partial least-squares discriminant analysis (OPLS-DA) was subsequently performed, and model validity and potential overfitting were evaluated using seven-fold cross-validation and 200 permutation tests. Differential metabolites were identified based on a variable importance in projection (VIP) score > 1 from the OPLS-DA model and a *p*-value < 0.05 from one-way analysis of variance (ANOVA).

### 2.8. Statistical Analysis

Statistical analyses were performed using SPSS Statistics 27.0 (IBM Corp., Armonk, NY, USA). Data are presented as the mean ± standard deviation (SD). Differences among groups were analyzed using one-way analysis of variance (ANOVA), followed by Tukey’s post hoc multiple-comparison test when appropriate. Differences were considered statistically significant at *p* < 0.05. Spearman’s correlation coefficients and corresponding *p*-values were calculated for each pairwise association. To account for multiple comparisons in the correlation analyses, *p*-values were adjusted using the Benjamini–Hochberg false discovery rate (FDR) procedure, and correlations with FDR-adjusted *p*-values (q-values) < 0.05 were considered statistically significant.

## 3. Result

### 3.1. Growth Performance

The growth performance of the lambs is presented in Table 2. Initial BW (body weight) did not differ among the CON, HC, and YC groups (*p* > 0.05). Compared with the CON group, both the HC and YC groups had greater final BW, total weight gain, and ADG (average daily gain) (*p* < 0.05), whereas no significant differences were observed between the HC and YC groups (*p* > 0.05). The YC group also showed greater ADFI (average daily feed intake) than the CON group (*p* < 0.05), while the HC group did not differ significantly from either the CON or YC group (*p* > 0.05). No significant differences were detected among groups for FCR (feed conversion ratio) or G (gain-to-feed ratio) (*p* > 0.05).

### 3.2. Changes in Rumen pH and Volatile Fatty Acids

The effects of dietary treatments on rumen pH and volatile fatty acid (VFA) concentrations are presented in Table 3. Relative to the CON group, the HC group showed a lower rumen pH (5.52 vs. 6.55), acetate concentration (39.00 vs. 50.94 mmol/L), butyrate concentration (5.54 vs. 6.95 mmol/L), and acetate-to-propionate ratio (1.31 vs. 2.67), together with higher propionate (30.09 vs. 19.15 mmol/L) and isobutyrate concentrations (1.07 vs. 0.77 mmol/L; *p* < 0.05). Yeast culture supplementation partially reversed these changes: compared with the HC group, the YC group had a higher rumen pH (6.32 vs. 5.52), acetate concentration (51.81 vs. 39.00 mmol/L), butyrate concentration (7.76 vs. 5.54 mmol/L), and acetate-to-propionate ratio (2.35 vs. 1.31), but a lower propionate concentration (22.14 vs. 30.09 mmol/L; *p* < 0.05). Moreover, acetate, butyrate, and the acetate-to-propionate ratio in the YC group were restored to levels comparable with those in the CON group (*p* > 0.05), although rumen pH remained lower in the YC group than in the CON group (6.32 vs. 6.55, *p* < 0.05). TVFA and isovalerate concentrations did not differ significantly among the three groups (*p* > 0.05).

### 3.3. Changes in Serum Biochemical Parameters

The effects of dietary treatments on serum biochemical parameters are presented in Table 4. Compared with the CON group, the HC group had lower serum TP (61.00 vs. 65.78 g/L), ALT (14.80 vs. 20.89 U/L), and AST (97.56 vs. 120.11 U/L) levels (*p* < 0.05). Compared with the HC group, serum ALT was higher in the YC group (21.41 vs. 14.80 U/L, *p* < 0.05) and did not differ significantly from that in the CON group (21.41 vs. 20.89 U/L, *p* > 0.05). Serum AST in the YC group was intermediate (103.98 U/L) between the CON (120.11 U/L) and HC (97.56 U/L) groups and did not differ significantly from either group. Serum TP remained lower in the YC group than in the CON group (60.15 vs. 65.78 g/L, *p* < 0.05). No significant differences were observed in GLU, ALB, ALP, UREA, UA, TG, or T-CHO among groups (*p* > 0.05).

### 3.4. Changes in Rumen Bacterial Community Diversity

**Figure 1:**
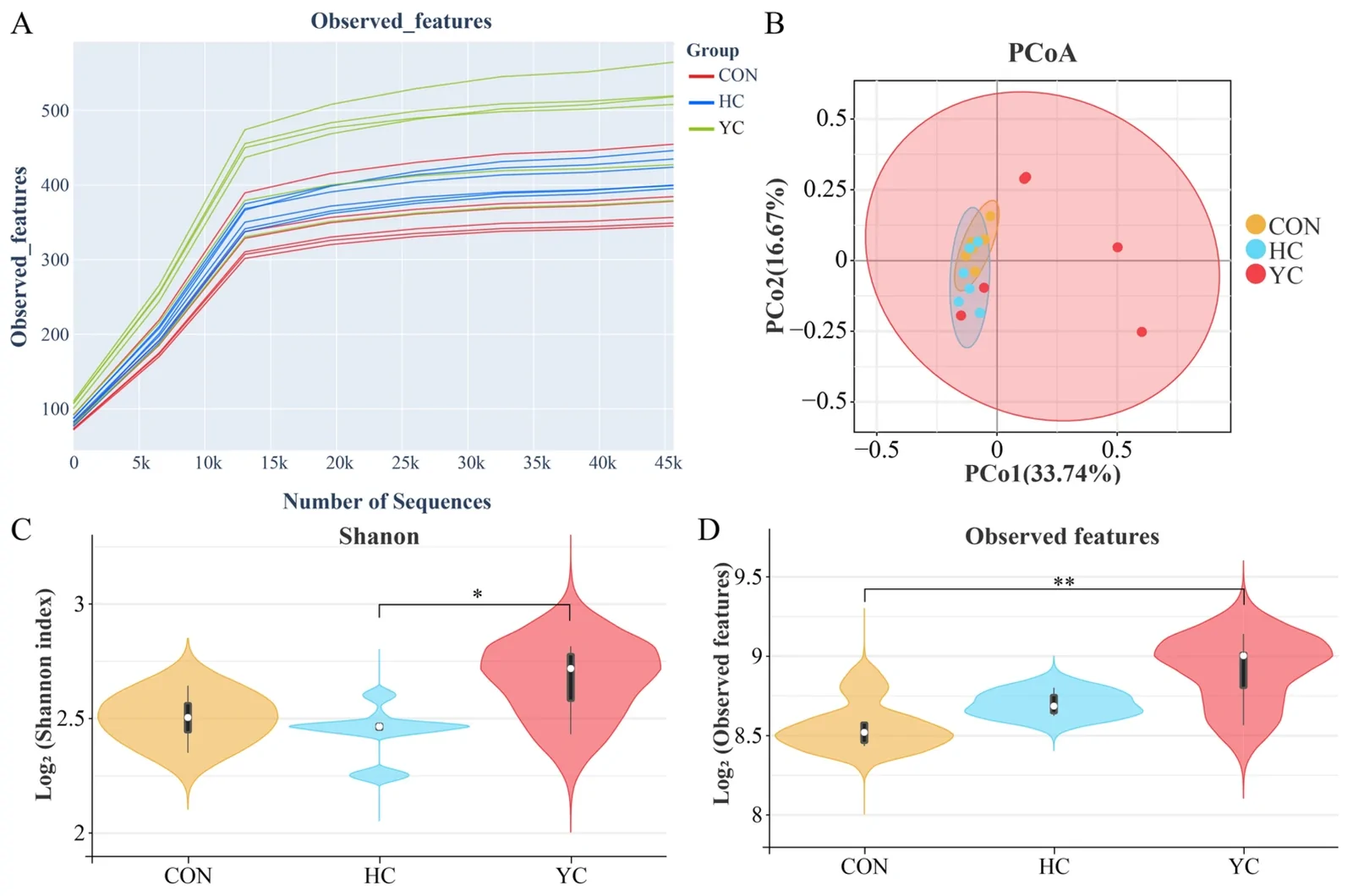
Effects of dietary treatments on sequencing depth and rumen bacterial community diversity in lambs. (**A**) Rarefaction curves based on observed features; (**B**) principal coordinate analysis (PCoA) based on Bray–Curtis distances; (**C**) Shannon index; (**D**) observed features. CON, control group; HC, high-concentrate diet group; YC, high-concentrate diet supplemented with 0.5% yeast culture group. * *p* < 0.05; ** *p* < 0.01.

As shown in Figure 1A, the rarefaction curves based on observed features gradually approached a plateau with increasing sequencing depth, indicating that the sequencing depth was sufficient to capture most of the bacterial richness in the rumen samples. Overall, the number of observed features was highest in the YC group, followed by the HC and CON groups.

Principal coordinate analysis (PCoA) based on Bray–Curtis distances showed that PCo1 and PCo2 explained 33.74% and 16.67% of the variation in bacterial community composition, respectively, accounting for 50.41% of the total variation (Figure 1B). The CON and HC samples showed partial overlap in the ordination space, whereas some YC samples tended to separate from those of the CON and HC groups. The YC samples also appeared to be more dispersed within the ordination space.

Alpha-diversity analysis showed that the Shannon index was significantly higher in the YC group than in the HC group (*p* < 0.05), whereas no significant differences were observed between the CON and HC groups or between the CON and YC groups (*p* > 0.05; Figure 1C). The number of observed features was significantly higher in the YC group than in the CON group (*p* < 0.01), whereas the HC group showed an intermediate value and did not differ significantly from either the CON or YC group (*p* > 0.05; Figure 1D). Additionally, Supplementary Table S1 presents further alpha-diversity metrics, showing that indices such as Chao1, Faith’s PD, Pielou’s evenness, and Good’s coverage.

### 3.5. Changes in Rumen Bacterial Community Composition and Candidate Differential Genera

**Figure 2:**
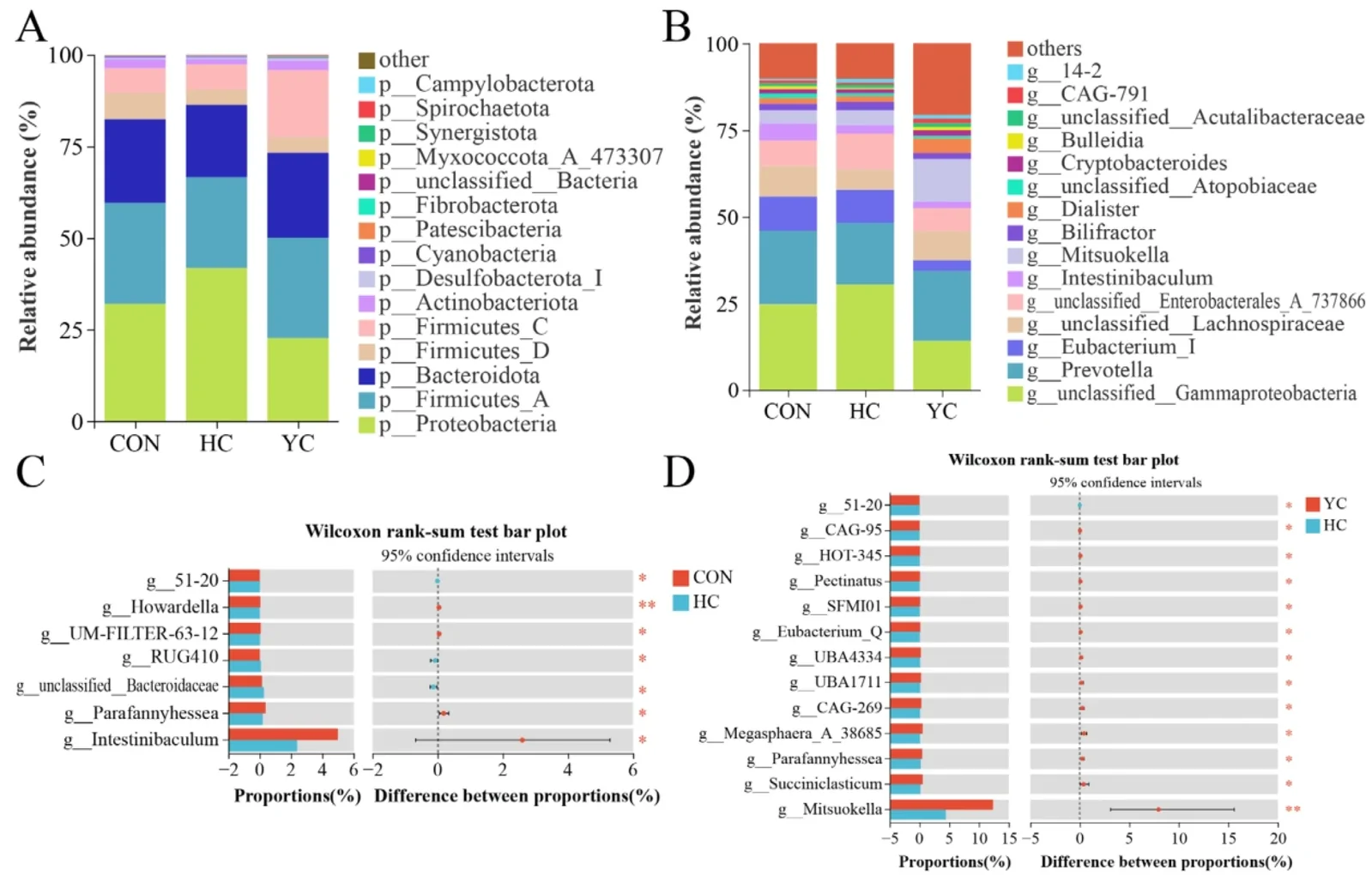
Effects of dietary treatments on rumen bacterial community composition and candidate differential genera in lambs. (**A**) Relative abundance of bacterial taxa at the phylum level; (**B**) relative abundance of predominant bacterial taxa at the genus level; (**C**) Wilcoxon rank-sum test of genus-level taxa between the CON and HC groups; (**D**) Wilcoxon rank-sum test of genus-level taxa between the HC and YC groups. CON, control group; HC, high-concentrate diet group; YC, high-concentrate diet supplemented with 0.5% yeast culture group. * *p* < 0.05; ** *p* < 0.01.

At the phylum level, Proteobacteria, Firmicutes_A, and Bacteroidota were the predominant phyla across all groups (Figure 2A). Compared with the CON group, the HC group showed a higher relative abundance of Proteobacteria and lower relative abundances of Firmicutes_A and Bacteroidota. In contrast, compared with the HC group, the YC group showed a lower relative abundance of Proteobacteria and higher relative abundances of Firmicutes_A, Bacteroidota, and Actinobacteriota.

At the genus level, the predominant genus-level taxa across the three groups included *g__unclassified_Gammaproteobacteria*, *g__Prevotella*, *g__Eubacterium_I*, *g__unclassified_Lachnospiraceae*, *g__Intestinibaculum*, and *g__Mitsuokella* (Figure 2B). Compared with the CON group, the HC group showed a higher relative abundance of *g__unclassified_Gammaproteobacteria* and lower relative abundances of *g__Prevotella*, *g__Intestinibaculum*, and *g__unclassified_Lachnospiraceae* (*p* < 0.05). Compared with the HC group, the YC group showed a lower relative abundance of *g__unclassified_Gammaproteobacteria* and higher relative abundances of *g__Prevotella*, *g__unclassified_Lachnospiraceae*, and *g__Mitsuokella* (*p* < 0.05).

Wilcoxon rank-sum tests identified seven genus-level taxa with nominal differences between the CON and HC groups (unadjusted *p* < 0.05; Figure 2C), including *g__51-20*, *g__Howardella*, *g__UM-FILTER-63-12*, *g__RUG410*, *g__unclassified_Bacteroidaceae*, *g__Parafannyhessea*, and *g__Intestinibaculum*. Among these taxa, *g__Howardella*, *g__Parafannyhessea*, and *g__Intestinibaculum* were more abundant in the CON group, whereas *g__51-20* and *g__unclassified_Bacteroidaceae* were more abundant in the HC group.

Similarly, 13 genus-level taxa showed nominal differences between the HC and YC groups (*p* < 0.05; Figure 2D), including *g__51-20*, *g__CAG-95*, *g__HOT-345*, *g__Pectinatus*, *g__SFMI01*, *g__Eubacterium_Q*, *g__UBA4334*, *g__UBA1711*, *g__CAG-269*, *g__Megasphaera_A_38685*, *g__Parafannyhessea*, *g__Succiniclasticum*, and *g__Mitsuokella*. Among these taxa, *g__Mitsuokella*, *g__Succiniclasticum*, *g__Megasphaera_A_38685*, *g__Parafannyhessea*, and *g__Pectinatus* were more abundant in the YC group than in the HC group, whereas *g__51-20* was less abundant. However, none of these differences remained statistically significant after FDR correction (*q* > 0.05). Therefore, these taxa were interpreted as potential YC-responsive genera rather than statistically confirmed differential taxa.

### 3.6. LEfSe Analysis of Differential Rumen Bacterial Taxa

**Figure 3:**
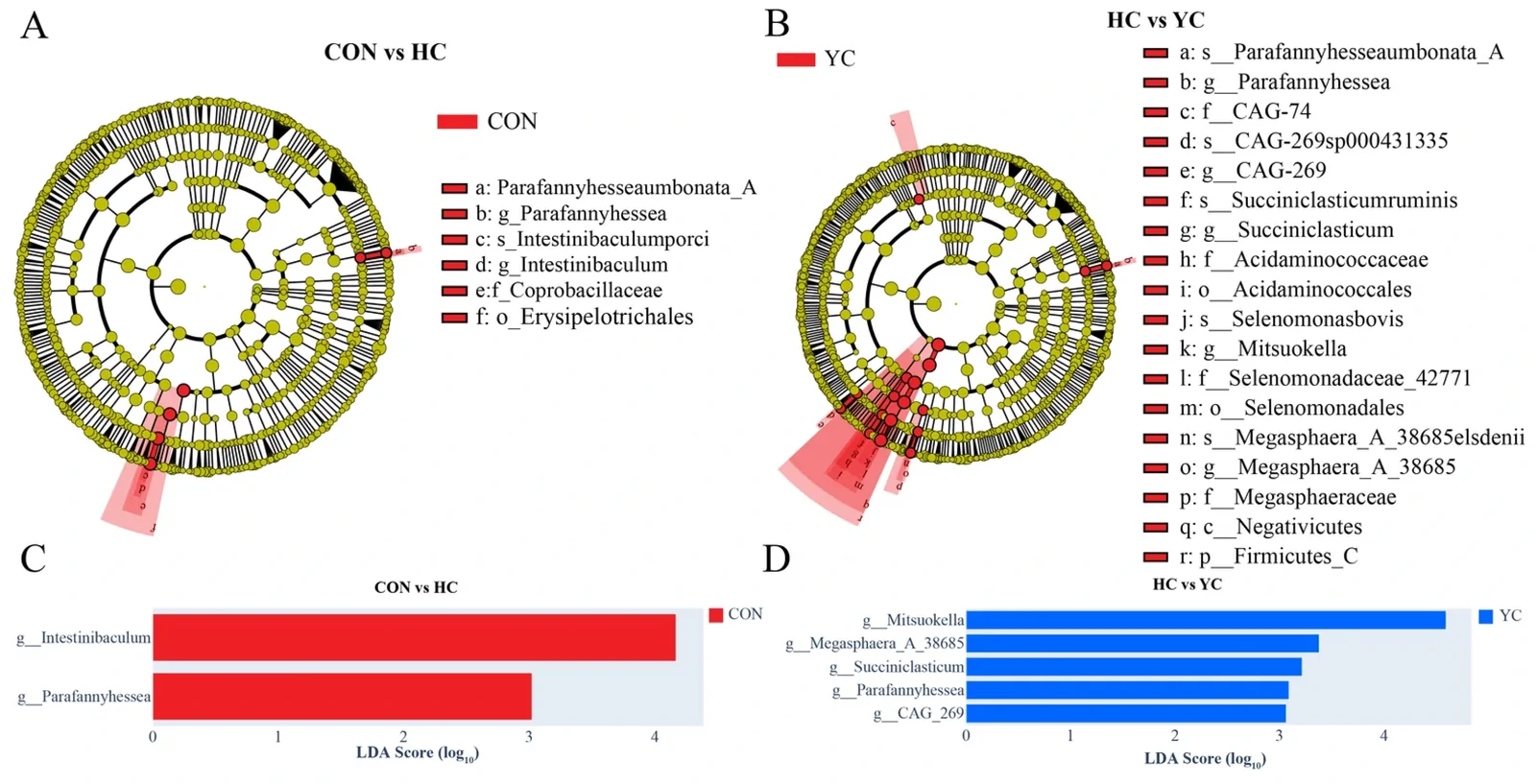
LEfSe analysis of differential rumen bacterial taxa under different dietary treatments. (**A**) LEfSe cladogram showing differential bacterial taxa between the CON and HC groups; (**B**) LEfSe cladogram showing differential bacterial taxa between the HC and YC groups; (**C**) LDA scores of discriminative genera between the CON and HC groups; (**D**) LDA scores of discriminative genera between the HC and YC groups. Different colors in the cladograms indicate taxa enriched in the corresponding groups, whereas yellow indicates taxa that did not differ significantly between groups. The bar plots show discriminative genera that exceeded the predefined LDA threshold.

LEfSe analysis revealed that the discriminatory bacterial taxa between the CON and HC groups were predominantly enriched in the CON group (Figure 3A,C). The LEfSe cladogram showed that these taxa were mainly distributed within clades including *o__Erysipelotrichales* and *f__Coprobacillaceae* and included *g__Intestinibaculum*, *s__Intestinibaculum_porci*, *g__Parafannyhessea*, and *s__Parafannyhessea_umbonata_A*. LDA scores further identified *g__Intestinibaculum* and *g__Parafannyhessea* as the major discriminative genera between the CON and HC groups, with *g__Intestinibaculum* exhibiting the highest LDA score. Under the predefined LEfSe screening criteria, no taxa enriched in the HC group met the threshold for discrimination.

Comparison between the HC and YC groups showed that the discriminatory taxa were predominantly enriched in the YC group (Figure 3B,D). These taxa were mainly distributed within clades including Firmicutes_C, *c__Negativicutes*, *o__Selenomonadales*, *f__Acidaminococcales*, and *f__Megasphaeraceae* and included *g__Mitsuokella*, *g__Megasphaera_A_38685*, *g__Succiniclasticum*, *g__Parafannyhessea*, and *g__CAG-269*. Among these genera, *g__Mitsuokella* had the highest LDA score, followed by *g__Megasphaera_A_38685*, *g__Succiniclasticum*, *g__Parafannyhessea*, and *g__CAG-269*. Using the same LEfSe criteria, no taxa enriched in the HC group met the predefined discrimination threshold.

### 3.7. Rumen Metabolic Profiles and Changes in Differential Metabolites

**Figure 4:**
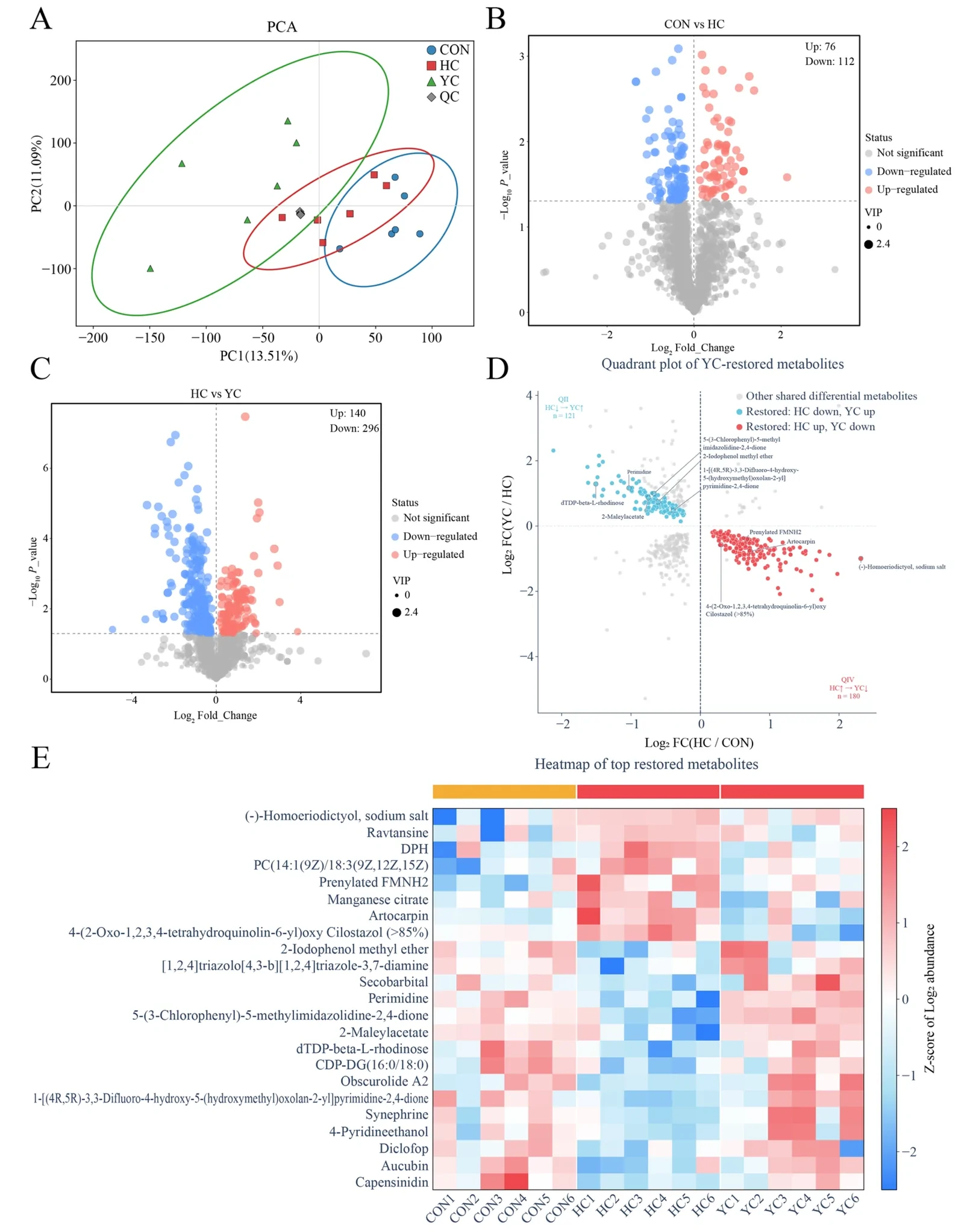
Effects of dietary treatments on rumen metabolic profiles and differential metabolites in lambs. (**A**) Principal component analysis (PCA) of rumen metabolic profiles in the CON, HC, and YC groups; (**B**) volcano plot of differential metabolites between the CON and HC groups; (**C**) volcano plot of differential metabolites between the HC and YC groups; (**D**) four-quadrant analysis of metabolites showing opposite directional changes following YC supplementation; (**E**) hierarchical clustering heatmap of representative metabolites showing opposite directional changes. CON, control group; HC, high-concentrate diet group; YC, high-concentrate diet supplemented with 0.5% yeast culture group. Ellipses denote 95% confidence intervals for each group.

Principal component analysis (PCA) revealed differences in the overall distribution of rumen metabolic profiles among the CON, HC, and YC groups (Figure 4A). PC1 and PC2 explained 13.51% and 11.09% of the total variance, respectively, accounting for 24.60% of the cumulative variance. The CON and HC samples partially overlapped in the PCA score plot, whereas the YC samples showed a tendency to separate from both the CON and HC samples. The QC samples clustered tightly, indicating good analytical stability and reproducibility.

Volcano plot analysis identified 188 differential metabolites between the CON and HC groups, of which 76 showed increased abundance and 112 showed decreased abundance (Figure 4B). Between the HC and YC groups, 436 differential metabolites were identified, of which 140 showed increased abundance and 296 showed decreased abundance (Figure 4C). The CON-versus-HC OPLS-DA model yielded R^2^Y(cum) = 0.992 and Q^2^(cum) = 0.040, whereas the HC-versus-YC model yielded R^2^Y(cum) = 0.998 and Q^2^(cum) = 0.694. The 200 permutation validation shows that the HC and YC models and the CON and HC models have good predictive robustness and can be analyzed later (Supplementary Figure S1).

Metabolites that differed between the HC and CON groups and showed an opposite directional change in the YC group relative to the HC group were further screened. Four-quadrant analysis showed that some metabolites that were decreased in the HC group relative to the CON group increased in the YC group relative to the HC group, whereas some metabolites that were increased in the HC group decreased following YC supplementation (Figure 4D). Hierarchical clustering of representative metabolites with opposite directional changes showed distinct abundance patterns across the three groups. For some metabolites, the abundance patterns in the YC group were more similar to those observed in the CON group than to those in the HC group (Figure 4E).

### 3.8. KEGG Pathway Enrichment of Differential Metabolites and Changes in Candidate Metabolites Showing Recovery Trends

**Figure 5:**
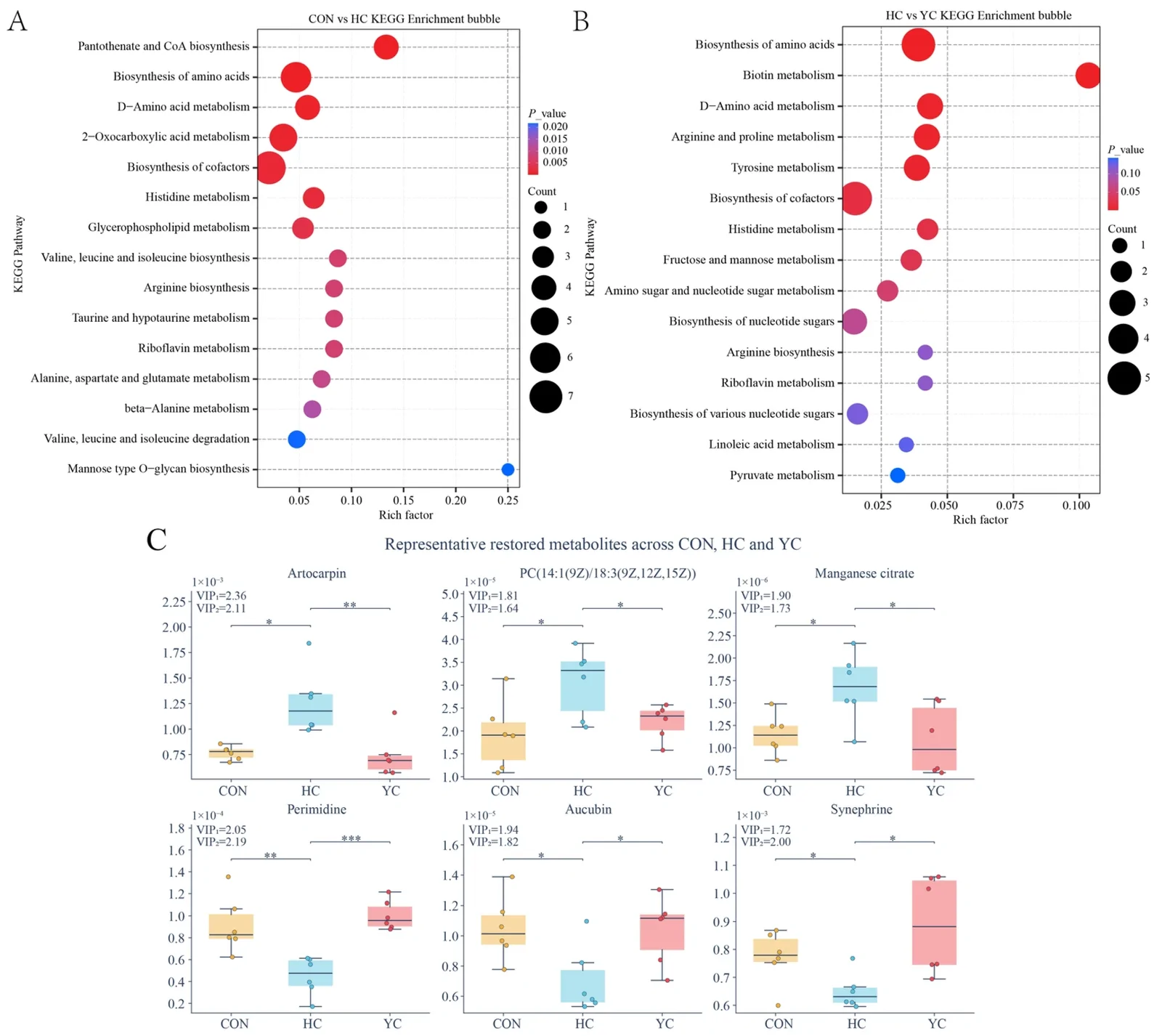
KEGG pathway enrichment of differential rumen metabolites and analysis of representative metabolites showing recovery trends under different dietary treatments. (**A**) KEGG pathway enrichment analysis of differential metabolites between the CON and HC groups; (**B**) KEGG pathway enrichment analysis of differential metabolites between the HC and YC groups; (**C**) relative levels of representative metabolites showing recovery trends in the CON, HC, and YC groups. Bubble size represents the number of differential metabolites mapped to the corresponding pathway, bubble color represents the enrichment *p*-value, and the rich factor represents the degree of enrichment of differential metabolites in each pathway. * *p* < 0.05, ** *p* < 0.01, *** *p* < 0.001.

KEGG pathway enrichment analysis showed that differential metabolites between the CON and HC groups were mainly enriched in pantothenate and CoA biosynthesis, biosynthesis of amino acids, D-amino acid metabolism, 2-oxocarboxylic acid metabolism, biosynthesis of cofactors, histidine metabolism, glycerophospholipid metabolism, and valine, leucine and isoleucine biosynthesis (Figure 5A). Differential metabolites between the HC and YC groups were mainly enriched in biosynthesis of amino acids, biotin metabolism, D-amino acid metabolism, arginine and proline metabolism, tyrosine metabolism, biosynthesis of cofactors, histidine metabolism, and fructose and mannose metabolism (Figure 5B). Several pathways, including biosynthesis of amino acids, D-amino acid metabolism, biosynthesis of cofactors, and histidine metabolism, were shared between the two comparisons, suggesting that both high-concentrate feeding and YC supplementation were associated with alterations in rumen amino acid- and cofactor-related metabolism.

Representative metabolites showing opposite directional changes between the HC-versus-CON and YC-versus-HC comparisons are presented in Figure 5C. The relative levels of artocarpin, PC(14:1(9Z)/18:3(9Z,12Z,15Z)), and manganese citrate were higher in the HC group than in the CON group but decreased following YC supplementation, shifting toward the levels observed in the CON group. Conversely, perimidine, aucubin, and synephrine were lower in the HC group than in the CON group but increased following YC supplementation, likewise shifting toward CON-group levels. All six metabolites had VIP scores > 1, and significant differences were observed in some pairwise comparisons, suggesting that they may represent candidate metabolites associated with the rumen metabolic response to YC supplementation under high-concentrate feeding conditions.

### 3.9. Correlation Analysis Among Rumen Bacterial Genera, Metabolites, and Phenotypic Parameters

**Figure 6:**
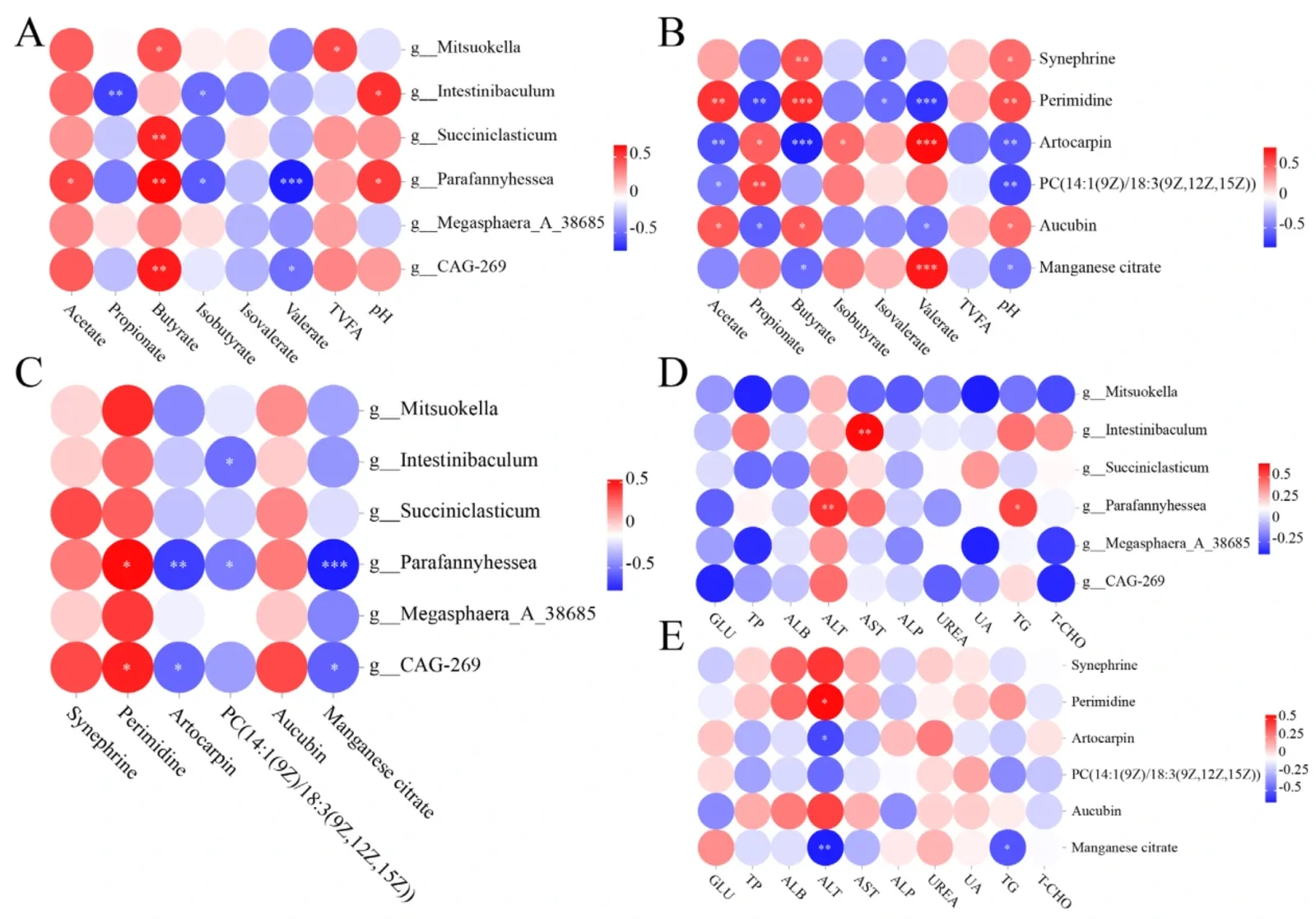
Correlation analysis among candidate YC-responsive rumen bacterial genera, representative metabolites showing recovery trends, rumen fermentation parameters, and serum biochemical parameters. (**A**) Correlations between candidate YC-responsive bacterial genera and rumen fermentation parameters; (**B**) correlations between representative metabolites showing recovery trends and rumen fermentation parameters; (**C**) correlations between candidate YC-responsive bacterial genera and representative metabolites showing recovery trends; (**D**) correlations between candidate YC-responsive bacterial genera and serum biochemical parameters; (**E**) correlations between representative metabolites showing recovery trends and serum biochemical parameters. Red indicates positive correlations, blue indicates negative correlations, and color intensity represents the strength of the correlations. * *p* < 0.05, ** *p* < 0.01, *** *p* < 0.001.

Correlation analysis revealed associations between several candidate rumen bacterial genera and rumen fermentation parameters (Figure 6A). Specifically, *g__Intestinibaculum* was significantly negatively correlated with propionate and isobutyrate and positively correlated with rumen pH. *g__Parafannyhessea* was significantly positively correlated with acetate, butyrate, and rumen pH, but negatively correlated with isobutyrate and valerate. Both *g__Succiniclasticum* and *g__CAG-269* were significantly positively correlated with butyrate, whereas *g__CAG-269* was significantly negatively correlated with valerate.

Representative metabolites showing recovery trends also exhibited distinct associations with rumen fermentation parameters (Figure 6B). Perimidine showed positive associations with acetate, butyrate, and rumen pH and negative associations with propionate, isovalerate, and valerate. Aucubin showed a similar pattern, with positive associations with acetate, butyrate, and rumen pH and negative associations with propionate and valerate. In contrast, artocarpin was significantly negatively correlated with acetate, butyrate, and rumen pH and positively correlated with propionate, isobutyrate, and valerate. PC(14:1(9Z)/18:3(9Z,12Z,15Z)) and manganese citrate exhibited partially similar correlation patterns.

Further analysis of associations between candidate bacterial genera and metabolites showing recovery trends revealed that *g__Parafannyhessea* was significantly positively correlated with perimidine and significantly negatively correlated with artocarpin, PC(14:1(9Z)/18:3(9Z,12Z,15Z)), and manganese citrate (Figure 6C). Similarly, *g__CAG-269* was significantly positively correlated with perimidine and significantly negatively correlated with artocarpin and manganese citrate. In addition, *g__Intestinibaculum* was significantly negatively correlated with PC(14:1(9Z)/18:3(9Z,12Z,15Z)).

Significant associations were also observed between candidate bacterial genera or representative metabolites showing recovery trends and serum biochemical parameters (Figure 6D,E). Among these associations, *g__Intestinibaculum* was significantly positively correlated with AST, whereas *g__Parafannyhessea* was significantly positively correlated with ALT and TG. Several metabolites showing recovery trends were also associated primarily with ALT and TG.

## 4. Discussion

High-concentrate diets provide large amounts of rapidly fermentable carbohydrates that are rapidly metabolized by rumen microorganisms, resulting in substantial organic acid production. When organic acid production exceeds the capacity for microbial utilization, epithelial absorption, and removal from the rumen, ruminal pH declines, increasing the risk of subacute ruminal acidosis (SARA) and potentially disrupting microbial homeostasis and fermentation [14,15]. Yeast culture contains a variety of fermentation-derived metabolites and bioactive compounds that may help stabilize the ruminal environment and improve fermentation efficiency [16]. In the present study, rumen pH was significantly lower in the HC treatment than in the CON treatment, whereas supplementation with 0.5% YC significantly increased rumen pH relative to the HC treatment, suggesting that YC partially alleviated ruminal acidification induced by high-concentrate feeding. In addition, the YC treatment showed a higher voluntary feed intake, as reflected by the increased average daily feed intake (ADFI) compared with the CON treatment, indicating that YC supplementation may also help maintain feed intake under high-concentrate feeding conditions. Wang et al. [17] similarly reported that supplementation of a starch-rich diet with 1% YC improved the rumen fermentation environment in lambs. At 6 h after feeding, rumen pH was higher in the YC treatment than in the control treatment (6.09 vs. 5.88), while total VFA and butyrate concentrations increased from 95.4 to 118 mmol/L and from 11.2 to 17.2 mmol/L, respectively. These findings are consistent with the present results and further support the capacity of YC to alleviate the decline in rumen pH and modulate fermentation under high-starch feeding conditions. In addition, when dairy cows were transitioned from a high-fiber to a high-concentrate diet, supplementation with active dry Saccharomyces cerevisiae (ADSC) reduced the duration for which rumen pH remained below 5.6 from 321 to 122 min/day during week 10 [18], further supporting the ability of yeast supplementation to stabilize ruminal pH under high-concentrate feeding conditions. High-concentrate feeding also significantly decreased acetate and butyrate concentrations and the acetate-to-propionate ratio, whereas YC supplementation significantly increased these parameters to levels that did not differ from those in the CON group. These findings suggest that YC not only attenuated ruminal acidification but also partially normalized the VFA fermentation pattern altered by high-concentrate feeding. A previous study reported that YC supplementation increased total VFA concentration from 77.13 to 97.90 mmol/L and acetate concentration from 44.47 to 57.29 mmol/L, while propionate and butyrate concentrations remained unchanged [19]. Xue et al. [20] reported that supplementation with 0.60‰ Saccharomyces cerevisiae significantly increased total VFA and acetate concentrations and the acetate-to-propionate ratio in heat-stressed goats. Similar improvements were also observed in the in vitro fermentation experiment. AIZahal et al. [18] reported that, during high-grain feeding, ADSC supplementation increased total ruminal VFA concentration from 154 ± 7.5 to 175 ± 7.5 mM and propionate from 94 ± 5.7 to 117 ± 6.1 mM. Sun et al. [21] and Song et al. [22] similarly found that increasing dietary corn from 350 to 600 g/kg tended to reduce serum ALT activity; in the unsupplemented treatments, ALT declined from 34.2 to 22.3 U/L. This pattern is consistent with the lower serum ALT observed in the HC treatment in the present study and suggests that high-concentrate feeding may alter enzymes associated with hepatic metabolism. Collectively, these responses may be associated with the ability of YC to create a more favorable ruminal environment for microbial activity and to facilitate the utilization of fermentation products, thereby contributing to greater stability of rumen fermentation under high-concentrate feeding conditions.

Serum biochemical parameters provide useful information on the nutritional and metabolic status of animals. In the present study, serum TP, ALT, and AST levels were significantly lower in the HC group than in the CON group, whereas ALP and T-CHO showed numerical increases and decreases, respectively. These changes suggest that high-concentrate feeding may influence systemic nutrient metabolism and the activities of enzymes associated with hepatic metabolism in lambs. Zhang et al. [23] compared sheep fed a low-grain diet (grain-to-forage ratio, 3:7; starch, 24.89%) with those fed a high-grain diet (7:3; starch, 38.64%). Under high-grain feeding, rumen pH remained below 5.6 for 1–4 h after feeding, while serum GSH and GPX4 decreased (*p* = 0.006 and *p* = 0.049, respectively), and hepatic Fe^2+^ and MDA increased (*p* = 0.036 and *p* = 0.018, respectively). The high-grain diet also increased hepatic ACSL4 expression (*p* < 0.001) and decreased GPX4 and SLC7A11 protein expression (*p* = 0.008 and *p* = 0.010, respectively), supporting an association between SARA, oxidative stress, and hepatic ferroptosis [23]. Song et al. [22] similarly found that increasing dietary corn from 350 to 600 g/kg tended to reduce serum ALT activity; in the unsupplemented treatments, ALT declined from 34.2 to 22.3 U/L. This pattern is consistent with the lower serum ALT observed in the HC treatment in the present study and suggests that high-concentrate feeding may alter enzymes associated with hepatic metabolism. Following supplementation with 0.5% YC, serum ALT levels increased significantly relative to the HC group and were comparable to those in the CON group. AST levels were intermediate between those of the CON and HC groups, whereas ALB and ALP did not differ significantly among the three groups. These findings indicate that the effects of YC on serum biochemical status were selective rather than uniform across all measured parameters. Previous studies have suggested that YC may influence systemic metabolic status by improving rumen fermentation and nutrient digestion and utilization [24,25], which is broadly consistent with the partial normalization of serum biochemical parameters observed in the present study. Together with the improvements in rumen pH and VFA profiles, these findings suggest that YC supplementation may contribute to maintaining metabolic homeostasis under high-concentrate feeding conditions, although the underlying systemic mechanisms require further investigation.

As the principal site of microbial fermentation in ruminants, the rumen harbors a diverse and complex microbial community whose composition and function are strongly influenced by dietary composition and nutritional management [26]. Modulation of the rumen microbiota may contribute to maintaining ruminal homeostasis and supporting host health [16]. In the present study, the Shannon index was significantly higher in the YC group than in the HC group, whereas the number of observed features was significantly higher in the YC group than in the CON group. These findings suggest that YC supplementation was associated with changes in rumen bacterial diversity and richness. Wang et al. [27] similarly reported that YC supplementation increased the Shannon index of the rumen bacterial community in sheep and altered microbial community composition. Notably, Gao et al. [9] reported that, in finishing cattle fed a high-concentrate diet, active dry yeast significantly increased alpha diversity, whereas yeast culture primarily affected the relative abundance of specific bacterial genera. These findings suggest that the effects of yeast-based additives on rumen microbial diversity may depend on the type and dose of the yeast preparation as well as the composition of the basal diet.

At the phylum level, Proteobacteria, Firmicutes_A, and Bacteroidota were the predominant bacterial phyla across all groups. Relative to the CON group, the HC group showed a higher relative abundance of Proteobacteria and lower relative abundances of Firmicutes_A and Bacteroidota. In contrast, the YC group showed a lower relative abundance of Proteobacteria and higher relative abundances of Firmicutes_A and Bacteroidota than the HC group. A previous study in goats reported that increasing the concentrate-to-forage ratio from 25:75 to 80:20 decreased Firmicutes (*p* = 0.006), Fibrobacteres (*p* = 0.004), Fibrobacter (*p* = 0.004), and *Ruminococcus flavefaciens* (*p* = 0.010), while increasing Proteobacteria (*p* < 0.010) [28]. *Ruminococcus flavefaciens* also decreased (*p* = 0.010), whereas *Prevotella* showed a numerical increase of 22.91% (*p* = 0.078) [28]. These shifts may reflect selective pressures imposed by greater starch availability and the accompanying decline in ruminal pH. Wang et al. [29] also reported that YC supplementation altered the relative abundances of predominant bacterial groups, including Bacteroidota and Proteobacteria. In the present study, YC supplementation partially restored rumen pH and shifted the VFA profile toward that observed in the CON group. Collectively, these findings suggest that YC may help mitigate high-concentrate diet-associated alterations in the rumen bacterial community, potentially contributing to a more stable ruminal microbial environment.

At the genus level, high-concentrate feeding was associated with lower relative abundances of *g__Intestinibaculum* and *g__Parafannyhessea*, whereas *g__Parafannyhessea*, *g__Succiniclasticum*, *g__Mitsuokella*, *g__Megasphaera_A_38685*, and *g__CAG-269* showed higher relative abundances in the YC group than in the HC group. These taxa may therefore represent potential YC-associated candidate genera related to changes in rumen fermentation under high-concentrate feeding conditions. *g__Parafannyhessea_umbonata* (formerly classified as *Olsenella_umbonata*), which was originally isolated from the ovine rumen, is an anaerobic bacterium capable of producing lactate [30]. In the present study, the relative abundance of *g__Parafannyhessea* was lower in the HC group but higher in the YC group, suggesting that YC supplementation may favor the persistence or recovery of this taxon under high-concentrate feeding conditions. *g__Intestinibaculum_porci*, a representative species of the genus *g__Intestinibaculum*, produces lactate as a major fermentation end product [31]. Its lower relative abundance in the HC group may reflect the sensitivity of this taxon to changes in substrate availability and the ruminal physicochemical environment induced by high-concentrate feeding.

Notably, *g__Mitsuokella*, *g__Succiniclasticum*, and *g__Megasphaera_A_38685* were enriched in the YC group. *Mitsuokella_jalaludinii*, originally isolated from the bovine rumen, can ferment carbohydrates to produce lactate, succinate, acetate, and other metabolites [32,33]. *Succiniclasticum_ruminis* is a characteristic rumen succinate-utilizing bacterium that converts succinate primarily to propionate [34]. *Megasphaera_elsdenii* is a well-characterized rumen lactate-utilizing bacterium capable of converting lactate into fermentation products including propionate and butyrate, thereby contributing to lactate removal under rapidly fermentable dietary conditions [35,36]. Although *M. elsdenii* is a well-characterized lactate-utilizing species within this broader taxonomic lineage, the specific metabolic capacity of *g__Megasphaera_A_38685* identified in the present study remains to be confirmed. Therefore, the enrichment of *g__Mitsuokella*, *g__Succiniclasticum*, and *g__Megasphaera_A_38685* in the YC group suggests that YC supplementation may have altered the production and cross-utilization of fermentation intermediates such as lactate and succinate. These microbial shifts may be associated with the higher rumen pH and altered VFA profile observed in the YC group. However, the relationships between individual bacterial taxa and final VFA concentrations are likely to be complex because ruminal VFA profiles reflect the combined effects of microbial production, cross-feeding, utilization, and absorption. The specific metabolic role of *g__CAG-269* in the rumen remains poorly characterized. A previous study in dairy cows reported that Clostridium sp. *g__CAG-269* was positively associated with propionate and total VFA concentrations but negatively associated with acetate, butyrate, the acetate-to-propionate ratio, and rumen pH [37]. In the present study, *g__CAG-269* was enriched in the YC group; however, its abundance pattern was not fully consistent with the changes observed in several VFA parameters. Therefore, the functional significance of *g__CAG-269* in the rumen response to YC supplementation remains to be clarified.

Untargeted metabolomic analysis showed that high-concentrate feeding markedly altered the rumen metabolic profile, whereas several metabolites shifted toward levels observed in the CON group following YC supplementation, suggesting that YC may partially alleviate high-concentrate diet-associated metabolic perturbations. Previous studies have shown that high-grain diets can markedly alter ruminal amino acid and lipid metabolism as well as microbial-derived metabolites, while YC supplementation can also modify rumen metabolic profiles under high-concentrate feeding conditions [9,27,38]. In the present study, the relative levels of synephrine, perimidine, and aucubin were lower in the HC group than in the CON group but increased following YC supplementation. Conversely, artocarpin, PC(14:1(9Z)/18:3(9Z,12Z,15Z)), and manganese citrate were higher in the HC group but decreased following YC supplementation, shifting toward the levels observed in the CON group. Aucubin and synephrine have been reported to possess antioxidant and anti-inflammatory activities [39,40]. Their shifts toward CON-group levels following YC supplementation may reflect broader changes in the rumen metabolic environment. PC(14:1(9Z)/18:3(9Z,12Z,15Z)), a phosphatidylcholine species, showed a response pattern consistent with alterations in glycerophospholipid metabolism identified in the pathway analysis, suggesting that both high-concentrate feeding and YC supplementation were associated with changes in rumen lipid metabolism [38]. Although artocarpin has been reported to exhibit antioxidant and anti-inflammatory activities [41], its higher relative level in the HC group suggests that its variation in the rumen may reflect a metabolic response to high-concentrate feeding rather than a straightforward indicator of beneficial metabolic status. In contrast, the biological roles of perimidine and manganese citrate in the rumen remain poorly characterized. Their consistent directional responses to YC supplementation therefore warrant further investigation as potential YC-responsive metabolites.

Correlation analysis further revealed associations between several candidate YC-responsive genera and rumen fermentation parameters. In the present study, *g__Parafannyhessea* was positively correlated with rumen pH, acetate, and butyrate, whereas *g__Intestinibaculum* was positively correlated with rumen pH, suggesting that these genera were closely associated with variations in the rumen fermentation environment. Smith et al. [42] reported a significant association between the relative abundance of *g__Intestinibaculum* and ruminal propionate concentration in finishing cattle, suggesting a potential link between this genus and rumen VFA metabolism. In the present study, *g__Succiniclasticum* was enriched in the YC group and was positively correlated with butyrate. This genus is closely associated with the ruminal succinate–propionate pathway because members of *g__Succiniclasticum* can utilize succinate as a substrate for propionate formation. Liu et al. [43] also reported that *g__Succiniclasticum* and *g__Prevotella* were strongly associated with propionate-related fermentation in the bovine rumen. Wang et al. [25] further reported that YC supplementation altered rumen fermentation and microbial community composition in sheep and that the relative abundances of *g__Megasphaera* and *g__Mitsuokella* were positively correlated with propionate concentration. Carpinelli et al. [44] also reported that yeast supplementation increased the abundance of the lactate-utilizing bacterium *Megasphaera_elsdenii* in dairy cows. Together, these observations suggest that YC-responsive bacterial taxa may be potentially associated with organic acid metabolism in the rumen. However, the relationship between the abundance of individual bacterial taxa and final VFA concentrations is not necessarily direct, because ruminal VFA profiles reflect the combined effects of microbial production, cross-feeding, utilization, and epithelial absorption. Associations between candidate bacterial genera and rumen metabolites further supported a potential link between microbial and metabolic responses to YC supplementation. In particular, *g__Parafannyhessea* was positively correlated with perimidine but negatively correlated with artocarpin, PC(14:1(9Z)/18:3(9Z,12Z,15Z)), and manganese citrate, whereas *g__CAG-269* was positively correlated with perimidine and negatively correlated with artocarpin and manganese citrate. A previous study in ewe hoggets reported that Parafannyhessea was negatively correlated with ruminal methane production, methane energy, and ammonia concentration [45]. In that study, ruminal methane production ranged from 35.8 to 36.7 g/d, methane energy from 1.98 to 2.03 MJ/d, and ammonia concentration from 59.1 to 65.5 mg/L across dietary treatments. These findings indicate exploratory associations between changes in selected bacterial genera and variations in rumen fermentation and metabolic profiles. However, the direct metabolic functions of *g__Parafannyhessea* and *g__CAG-269* in the rumen remain poorly characterized, and their functional roles in the response to YC supplementation require further investigation.

Several limitations of this study should be acknowledged. Although 15 lambs were included in each dietary treatment during the feeding trial, only six animals per group were subjected to terminal sampling for serum biochemical, rumen fermentation, 16S rRNA gene sequencing, and untargeted metabolomic analyses. The relatively small number of biological replicates limits the statistical power to detect modest treatment effects and may increase the susceptibility of high-dimensional microbiome and metabolome analyses to unstable estimates and false-positive findings. Therefore, taxa and metabolites identified in the present study should be interpreted primarily as candidate YC-responsive features rather than definitive biomarkers or causal mediators. We attempted to reduce this risk by treating individual animals as biological replicates, applying multiple-testing correction where applicable, emphasizing consistent patterns across fermentation, microbiome, and metabolome datasets, and avoiding causal interpretation of correlation analyses. Nevertheless, validation in larger independent cohorts and, where appropriate, targeted microbiological or metabolomic assays will be necessary to confirm these findings.

High-concentrate feeding reduced rumen pH, altered the VFA profile, and was accompanied by changes in rumen bacterial community composition and metabolic profiles, whereas supplementation with 0.5% YC partially alleviated these alterations. Correlation analysis further revealed significant associations of candidate YC-responsive genera (*g__Mitsuokella*, *g__Intestinibaculum*, *g__Succiniclasticum*, *g__Parafannyhessea*, *g__Megasphaera_A_38685*, and *g__CAG-269*) and metabolites showing recovery trends (synephrine, perimidine, artocarpin, PC(14:1(9Z)/18:3(9Z,12Z,15Z)), aucubin, and manganese citrate) with rumen fermentation parameters and selected serum biochemical parameters. These associations suggest that YC supplementation was accompanied by coordinated changes in rumen bacterial community composition, metabolic profiles, and fermentation characteristics. However, the present correlation analyses are exploratory and cannot establish causal relationships among these variables.

## 5. Conclusions

This study showed that high-concentrate feeding significantly reduced rumen pH, altered the VFA profile, and was associated with changes in rumen bacterial community composition and metabolic profiles in lambs. Supplementation with 0.5% YC significantly increased rumen pH, acetate and butyrate concentrations, and the acetate-to-propionate ratio, while decreasing propionate concentration relative to the HC group. YC supplementation was also associated with changes in bacterial richness and diversity and with shifts in selected candidate genera, including *g__Mitsuokella*, *g__Megasphaera_A_38685*, and *g__Succiniclasticum*. Metabolomic analysis further revealed marked alterations in the rumen metabolic profile following YC supplementation, particularly in pathways related to amino acid and cofactor metabolism, while several metabolites altered by high-concentrate feeding shifted toward levels observed in the CON group. Correlation analysis further showed that several candidate YC-responsive genera and metabolites showing recovery trends were significantly associated with rumen pH, VFA concentrations, and selected serum biochemical parameters. Overall, supplementation with 0.5% YC partially alleviated ruminal acidification, as reflected by attenuation of the pH decline and partial normalization of the VFA profile. These findings suggest that the beneficial effects of YC are associated with coordinated changes in rumen bacterial community composition and metabolic profiles, which may contribute to the maintenance of rumen microbial and metabolic homeostasis under high-concentrate feeding conditions.

## Figures and Tables

**Table 1:** Compositions and nutrient levels of the diets.

Items (%)	Groups		
	CON	HC	YC
Alfalfa hay meal	43.50	37.00	37.00
Corn	26.90	39.50	39.00
Soybean hulls	6.20	2.10	2.10
Sprayed corn bran	1.60	–	–
Corn germ meal	–	10.00	10.00
Wheat flour	5.00	4.60	4.60
Wheat bran	12.00	–	–
Soybean meal	–	1.60	1.60
Sugarcane molasses	2.00	2.00	2.00
Expanded urea	0.30	0.30	0.30
Limestone powder	0.10	0.30	0.30
Dicalcium phosphate	0.90	1.10	1.10
Sodium chloride	0.50	0.50	0.50
Yeast culture	–	–	0.50
Premix ^1^	1.00	1.00	1.00
Total	100.00	100.00	100.00
Nutrient			
CP, % DM	14.63	14.59	14.69
EE, % DM	2.69	2.71	2.69
Starch, % DM	26.55	34.97	34.64
NDF, % DM	30.54	24.98	25.02
ADF, % DM	19.13	15.28	15.32
Ca, % DM	0.89	0.90	0.90
Total P, % DM	0.50	0.49	0.49
Ca:P	1.80:1	1.83:1	1.83:1
ME, MJ/kg DM	10.78	11.39	11.39

Note: CON, control group; HC, high-concentrate diet group; YC, high-concentrate diet supplemented with 0.5% yeast culture group. DM, dry matter; CP, crude protein; EE, ether extract; NDF, neutral detergent fiber; ADF, acid detergent fiber; ME, metabolizable energy. ^1^ The premix provided the following per kg of diet: VA 15 000 IU, VD 2 200 IU, VE 50 IU, Fe 55 mg, Cu 12.5 mg, Mn 47 mg, Zn 24 mg, Se 0.5 mg, I 0.5 mg, Co 0.1 mg.

**Table 2:** Effects of yeast culture supplementation on the growth performance of lambs fed a high-concentrate diet. (*n* = 15).

Item	CON	HC	YC	SEM	*p*-Value
Initial BW, kg	21.43	21.31	21.37	0.101	0.730
Final BW, kg	37.83 ^b^	39.38 ^a^	39.27 ^a^	0.225	0.005
Total weight gain, kg	16.40 ^b^	18.07 ^a^	17.91 ^a^	0.318	0.018
ADG, g/d	287.72 ^b^	316.96 ^a^	314.15 ^a^	5.584	0.018
ADFI, kg/d	1.741 ^b^	1.795 ^ab^	1.840 ^a^	0.017	0.019
FCR, feed/gain	6.05	5.66	5.86	0.109	0.114
G:F, gain/feed	0.165	0.177	0.171	0.003	0.108

Note: Values are presented as means. CON, control group; HC, high-concentrate diet group; YC, high-concentrate diet supplemented with 0.5% yeast culture group; SEM, standard error of the mean; BW, body weight; ADG, average daily gain; ADFI, average daily feed intake; FCR, feed conversion ratio; G:F, gain-to-feed ratio. Different lowercase letters within the same row indicate statistically significant differences between treatments (*p* < 0.05), whereas the same letter indicates no significant difference (*p* > 0.05).

**Table 3:** Effects of different diet treatments on lamb rumen pH and volatile fatty acids (*n* = 6).

Indicators	CON	HC	YC	SEM	*p*-Value
pH	6.55 ^a^	5.52 ^c^	6.32 ^b^	0.033	<0.001
TVFA (mmol/L)	80.28 ^a^	78.67 ^a^	85.09 ^a^	1.820	0.062
Acetate (mmol/L)	50.94 ^a^	39.00 ^b^	51.81 ^a^	1.223	<0.001
Propionate (mmol/L)	19.15 ^c^	30.09 ^a^	22.14 ^b^^c^	0.979	<0.001
Butyrate (mmol/L)	6.95 ^a^	5.54 ^b^	7.76 ^a^	0.258	<0.001
Isobutyrate (mmol/L)	0.77 ^c^	1.07 ^a^	0.89 ^b^^c^	0.040	<0.001
Isovalerate (mmol/L)	1.08 ^a^	1.36 ^a^	1.21 ^a^	0.064	0.025
Valerate (mmol/L)	1.39 ^ab^	1.60 ^a^	1.27 ^b^	0.064	0.008
A/P	2.67 ^a^	1.31 ^b^	2.35 ^a^	0.094	<0.001

Note: Values are presented as means. CON, control group; HC, high-concentrate diet group; YC, high-concentrate diet supplemented with 0.5% yeast culture group; SEM, standard error of the mean; TVFA, total volatile fatty acids; A/P, acetate-to-propionate ratio. Different lowercase letters within the same row indicate statistically significant differences between treatments (*p* < 0.05), whereas the same letter indicates no significant difference (*p* > 0.05).

**Table 4:** Effects of different lean-to-coarse feed ratios and yeast cultures on serum biochemical indicators of lambs (*n* = 6).

Indicators	CON	HC	YC	SEM	*p*-Value
GLU (mmol/L)	7.29 ^a^	7.55 ^a^	7.06 ^a^	0.234	0.366
TP (g/L)	65.78 ^a^	61.00 ^b^	60.15 ^b^	1.063	0.004
ALB (g/L)	32.90 ^a^	31.18 ^a^	32.09 ^a^	0.539	0.112
ALT (U/L)	20.89 ^a^	14.80 ^b^	21.41 ^a^	0.741	<0.001
AST (U/L)	120.11 ^a^	97.56 ^b^	103.98 ^ab^	5.248	0.023
ALP (U/L)	526.73 ^a^	641.89 ^a^	532.20 ^a^	45.010	0.159
UREA (mmol/L)	22.75 ^a^	20.82 ^a^	21.33 ^a^	1.138	0.481
UA (μmol/L)	18.53 ^a^	19.02 ^a^	16.57 ^a^	2.359	0.744
TG (mmol/L)	0.46 ^a^	0.41 ^a^	0.41 ^a^	0.032	0.434
T-CHO (mmol/L)	2.22 ^a^	1.98 ^a^	1.95 ^a^	0.078	0.058

Note: Values are presented as means. CON, control group; HC, high-concentrate diet group; YC, high-concentrate diet supplemented with 0.5% yeast culture group; SEM, standard error of the mean; GLU, glucose; TP, total protein; ALB, albumin; ALT, alanine aminotransferase; AST, aspartate aminotransferase; ALP, alkaline phosphatase; UREA, urea; UA, uric acid; TG, triglycerides; T-CHO, total cholesterol. Different lowercase letters within the same row indicate statistically significant differences between treatments (*p* < 0.05), whereas the same letter indicates no significant difference (*p* > 0.05).

## Data Availability

This study generated a total of four sets of sequencing data; this paper analyzes three of them: CON, HC, and YC. All raw data have been submitted to the NCBI SRA database under accession number PRJNA1514914.

## Supplementary Materials

The following supporting information can be downloaded at https://www.mdpi.com/article/10.3390/microorganisms14092091/s1, Supplementary Table S1. Alpha diversity indices of the rumen microbial communities in different treatment groups; Supplementary Figure S1. OPLS-DA score plots and permutation validation of rumen metabolomic profiles.
